# 663 Evaluating the Impact of a Burn Image-Sharing App on Transfer Appropriateness and Efficiency

**DOI:** 10.1093/jbcr/iraf019.292

**Published:** 2025-04-01

**Authors:** Sachi Shinde, Cole Walker, Jamie Hollowell, Lori Chrisco, C Miller, Booker King, Sharon Schiro

**Affiliations:** University of North Carolina Jaycee Burn Center; Brody School of Medicine at East Carolina University; University of North Carolina Jaycee Burn Center; University of North Carolina Jaycee Burn Center; University of North Carolina Jaycee Burn Center; University of North Carolina Jaycee Burn Center; University of North Carolina at Chapel Hill

## Abstract

**Introduction:**

Burn injuries are a major public health concern, leading to about 500,000 emergency department (ED) visits annually in the U.S. Many smaller facilities without specialized burn care often refer these patients to burn centers. Although the number of burn injuries is on the rise in recent years, they remain relatively rare, resulting in limited experience among providers at non-burn centers. This lack of experience often leads to over-triaging, unnecessary transfers, higher costs, and resource strain. Recent studies have explored the use of telemedicine and photography to improve triage and reduce unnecessary transfers. By sharing patient history and images of burns, referring providers can make more informed decisions about whether to transfer a patient. The purpose of this study was to evaluate the effectiveness of an image-sharing app in increasing the appropriateness of transfers to a burn center.

**Methods:**

A burn image-sharing app is being piloted at selected referring facilities to facilitate transfers to a regional burn center. To assess its effectiveness, data from the burn center’s burn registry were analyzed. The study compared two referring hospitals—one using the app and one not—chosen for their similar patient and hospital profiles. The data analyzed included consultation times for transfer decisions, total burn surface area (TBSA), length of hospital stay, and patient age group. Institution A does not have access to the app, but Institution B does at this time.

**Results:**

Forty patients were included, 20 from Institution A (not using the app) and 20 from Institution B (using the app). The average transfer time at Institution A was 37.7 minutes compared to 84.3 minutes (p=0.2000) at Institution B. The average TBSA for Institution A was 6.7% versus 3.5% at Institution B (p=0.1200). Length of stay was similar: 5.25 days at Institution A and 4.26 days at Institution B (p=0.6248). Age group analysis revealed that geriatric patients at Institution A had a higher TBSA (14.1% vs. 4.8%) and longer evaluation times (61.8 vs. 22 minutes), possibly due to the complexity of assessing burns in older patients. Qualitative reviews by burn advanced practice providers indicated that burn image information augmented their transfer decisions but was not always the dominant factor in the decision.

**Conclusions:**

This study suggests that burn image-sharing apps can improve the efficiency of transfer decisions. The differences noted between the two institutions, while not significant, were likely due to the small N in our preliminary study and suggest the need for additional data.

**Applicability of Research to Practice:**

This study highlights the potential of telemedicine to enhance burn care and reduce unnecessary transfers. Further data collection is needed to refine the app and assess additional parameters that could streamline the transfer process more effectively.

**Funding for the Study:**

N/A

